# Continental divide: Predicting climate-mediated fragmentation and biodiversity loss in the boreal forest

**DOI:** 10.1371/journal.pone.0176706

**Published:** 2017-05-15

**Authors:** Dennis L. Murray, Michael J. L. Peers, Yasmine N. Majchrzak, Morgan Wehtje, Catarina Ferreira, Rob S. A. Pickles, Jeffrey R. Row, Daniel H. Thornton

**Affiliations:** 1 Department of Biology, Trent University, Peterborough, ON, Canada; 2 Department of Biological Sciences, University of Alberta, Edmonton, Alberta, Canada; 3 Department of Conservation Biology, UFZ–Helmholtz Centre for Environmental Research, Leipzig, Germany; 4 Panthera, New York, NY, United States of America; 5 Environment and Resource Studies, University of Waterloo, Waterloo, Ontario, Canada; 6 School of Environment, Washington State University, Pullman, WA, United States of America; Pacific Northwest National Laboratory, UNITED STATES

## Abstract

Climate change threatens natural landscapes through shifting distribution and abundance of species and attendant change in the structure and function of ecosystems. However, it remains unclear how climate-mediated variation in species’ environmental niche space may lead to large-scale fragmentation of species distributions, altered meta-population dynamics and gene flow, and disrupted ecosystem integrity. Such change may be especially relevant when species distributions are restricted either spatially or to a narrow environmental niche, or when environments are rapidly changing. Here, we use range-wide environmental niche models to posit that climate-mediated range fragmentation aggravates the direct effects of climate change on species in the boreal forest of North America. We show that climate change will directly alter environmental niche suitability for boreal-obligate species of trees, birds and mammals (*n* = 12), with most species ranges becoming smaller and shifting northward through time. Importantly, species distributions will become increasingly fragmented, as characterized by smaller mean size and greater isolation of environmentally-suitable landscape patches. This loss is especially pronounced along the Ontario-Québec border, where the boreal forest is narrowest and roughly 78% of suitable niche space could disappear by 2080. Despite the diversity of taxa surveyed, patterns of range fragmentation are remarkably consistent, with our models predicting that spruce grouse (*Dendragapus canadensis)*, boreal chickadee (*Poecile hudsonicus*), moose (*Alces americanus*) and caribou (*Rangifer tarandus*) could have entirely disjunct east-west population segments in North America. These findings reveal potentially dire consequences of climate change on population continuity and species diversity in the boreal forest, highlighting the need to better understand: 1) extent and primary drivers of anticipated climate-mediated range loss and fragmentation; 2) diversity of species to be affected by such change; 3) potential for rapid adaptation in the most strongly-affected areas; and 4) potential for invasion by replacement species.

## Introduction

Mounting evidence supports widespread concern that the earth’s biomes are facing rapid and increasingly profound stress associated with climate change [1]. Global trends attributable to climate change include pole-ward (or elevational) range shifts, phenological advances in growth or reproduction, and changes in abundance and community structure [2–4]. Most recently, these trends appear to be accelerating across a range of taxa and systems [5,6], leaving little doubt that the present course of environmental change will dramatically alter the structure and function of all ecosystems on earth. Yet, our understanding of biological responses to climate change often is obfuscated by non-climatic stressors that also affect habitat loss and range decline but tend to occur at smaller spatial or temporal scales [7]. Indeed, it remains challenging to infer or predict ecosystem- or biome-level responses to environmental change in the absence of more comprehensive investigation addressing large-scale multi-species patterns, and a greater focus on identifying commonalities in response of diverse taxa having broadly different interactions with their environment [8]. Further, a robust understanding of climate change responses is especially crucial for species that either occupy restricted environmental niche space, or else are found in marginal or rapidly-changing environments, as these groups can be among those most severely impacted by climate change [9]. Accordingly, broad-scale and multispecies analyses are necessary to better reveal the likely breadth and extent of climate-driven processes occurring through space and time.

Climate change can directly restrict the environmental niche of a species, leading to range loss especially in peripheral regions of the distribution [10]. This loss can be offset, to some extent, by northward shifts in distribution [11], but normally there are limits to how far or how quickly species ranges can respond to environmental change (e.g., [12]). In theory, climate change also may break apart niche space into smaller patches (i.e., *fragmentation*, sensu [13]) with lower suitability due to smaller size, stronger edge effects, and higher spatial isolation from other suitable patches. It follows that climate-mediated fragmentation may alter the spatial configuration of species distributions by creating checkerboard or disconnected distributions, thereby affecting meta-population dynamics and gene flow [14,15]; these effects can compound those from climate-mediated range loss and shift, and may be most substantive where distribution and abundance are limited, dispersal is restricted, or at the confluence of extreme or rare environments. Accordingly, the process of climate-mediated fragmentation may be akin to the known effects of anthropogenic habitat fragmentation on species distribution and occupancy, although an important distinguishing feature is that climate-mediated fragmentation may not create either the hard edges or instantaneous transformation that typically characterize most cases of anthropogenic habitat fragmentation. Further, although effects of habitat fragmentation on species ranges may be considerably less than those of direct habitat loss [16], to date, the potential role of climate on species range fragmentation and attendant changes in meta-population dynamics and gene flow, range continuity, and ecosystem integrity, has not been widely investigated.

The boreal forest biome of North America, which spans the Pacific-to-Atlantic coasts and covers roughly 6 million square kilometers across much of Canada, Alaska, and parts of the northern continental United States, is the largest, mostly intact, terrestrial ecosystem in the world [17]. The boreal forest is home to a variety of unique species and communities, and is subject to a range of important threats, of which climate change is perhaps the most important [18–20]. Recent shifts in temperature and precipitation in the boreal region have caused reduced snow cover, loss of permafrost, extended growing seasons, and severe drought [21,22]. Likewise, surges in the incidence of disease, fire, hybridization, and invasive species colonization are further consequences of climate change in the boreal forest [23–25]. Such changes have caused discernible alteration in the local or regional distribution and abundance of boreal plants and animals, including range displacement, altered dispersion patterns, increased spatial heterogeneity, and shifts in numerical trends [19,26,27]. Yet, there remains considerable uncertainty in the spatiotemporal extent and consistency of climate-mediated distributional change among boreal species, which reflects the extensive geographic range of the biome itself and the difficulty in effectively tracking changes at such a large scale and across many diverse species. Accordingly, we lack a robust understanding of the magnitude and extent of climate-mediated changes that are forthcoming to the boreal forest, leaving us in a precarious position regarding suitable conservation or management strategies for addressing likely impacts [28,29].

The current extent of the North American boreal biome reaches its narrowest region immediately south of James Bay, along the Ontario-Québec border. Here, the total width of the boreal forest is <500 km between James Bay and the transitional mixed forests to the south. This region coincides with the confluence of the North Atlantic Oscillation and Pacific Decadal Oscillation climate phenomena, and this region seems to be especially vulnerable to the effects of climate change [30,31]. Already, climate differentiation in the region appears to contribute to patterns of isolation between some populations [14,15], potentially leading to substantive deterioration of the boreal forest in that region. That this region coincides with the narrowest extent of the boreal forest in North America reveals potential vulnerability in maintaining transcontinental connectivity among populations of boreal-obligate species.

Here, we project climate suitability for 12 boreal-obligate tree, mammal, and bird species, to assess the extent of potential future climate-mediated loss and fragmentation of environmental niche space. We modeled species’ environmental niche according to current, 2050 and 2080 climate projections, across the entire biome. We then assessed patterns of climate-mediated fragmentation of environmental suitability, with focus on the Ontario-Québec bottleneck region. We predicted that decline in climate suitability would be most severe in this region, causing similar responses among a variety of boreal-obligate species, and potentially leading to disjunct east-west population segments and overall decline in species richness.

## Materials and methods

### a) Ecological niche modelling

We modeled current and future environmental suitability of 4 tree species, 3 bird species, and 5 mammal species; these species are recognized as being boreal-obligate, with several also serving as keystone, sentinel, or umbrella species in the biome [29,32]. Species were selected for our analysis specifically on the basis that: i) they were year-round residents of the boreal forest; and ii) a large percentage of their distribution (>70%) occurred within the boreal forest [29]. Notably, our criteria excluded migratory species, as well as those having more generalized habitat requirements that extended substantially outside the boreal forest. We also excluded Canada lynx (*Lynx canadensis*), which met our criteria but whose distribution closely matches that of snowshoe hares (*Lepus americanus*), their primary prey [33]. Note that we previously examined potential lynx population connectivity relative to climate change [15].

The program MaxEnt was used to model current and future environmental suitability (i.e., relative probability of occurrence) for each boreal forest species. MaxEnt is a presence-background approach which compares presence records with a random sample of background pixels from the covariates to develop a projection of climate suitability and determine the effect of environmental variables on species presence [34]. This is a machine learning approach used to make inference from incomplete information which is often the case using presence-only data [34,35]. We chose MaxEnt to project future range-wide distributions because data that are available at the spatial scale of interest are presence-only, and MaxEnt is recognized as an effective approach for modelling such datasets [36,37]. For example, predicted species’ range limits derived from MaxEnt models are known to correspond well with demographic constraints on population growth and persistence [38,39], which highlights the close association between environmental niche models and biological processes underlying species distributions. We collected presence records for boreal species across North America (see Table A, Figure A in S1 File) using the Global Biodiversity Information Facility (GBIF: www.gbif.org). GBIF records for caribou (*Rangifer tarandus*) were limited and we supplemented these observations with records derived from a variety of additional sources [40]. We removed data collected prior to 1950 to match environmental data (see below). To address outliers, we removed presence records that fell outside 100km from the recognized species range, as determined by the IUCN (www.iucnredlist.org) and BirdLife International (www.birdlife.org).

Climatic variables were obtained from the WorldClim database spanning 1950–2000 [41]. Given the large number of potential environmental variables, we included sets of bioclimatic variables that we thought a priori would be biologically relevant [33], and these environmental data were resampled to 10 X 10 km grid cell size. In total, eight bioclimatic variables were included in the final MaxEnt modeling (annual mean temperature, maximum temperature of the warmest month, minimum temperature of the coldest month, temperature seasonality, annual precipitation, precipitation seasonality, precipitation of the wettest quarter, and precipitation of the driest quarter, see Figure C in S1 File).

We used two general circulation models (GCM): the Canadian Centre for Climate Modelling and Analysis model CGCM3, and the Commonwealth Scientific and Industrial Research Organization model CSIRO mk3.5. Under each GCM we used the A2 scenario to forecast suitability into the future. Notably, the A2 scenario is the most liberal climate projection, but it is increasingly seen as the best reflection of current patterns in global carbon emissions [42,43]. In addition, while more up-to-date climate models currently exist, their performance is qualitatively similar to the models used herein (see [44]), allowing us to examine patterns of climate fragmentation irrespective of the specific climate model. Downscaled climate grids in bioclim format for 2050 and 2080 for each GCM and each scenario were downloaded from the Climate Change, Agriculture and Food Security (CCAFS) website (www.ccafs-climate.org). All future projections were resampled using cubic convolution in ArcGIS 10 to 10 X 10 km grid cell size to match the current environmental data.

Presence records obtained from GBIF or similar sources can be biased given collection patterns favouring sites closer to human infrastructure, such as roads [45]. The potential bias in presence records was addressed by creating a target-group background for use in MaxEnt modeling, following procedures outlined in [46]. In our models, GBIF records from 1950–2000 for the family or order of each species (depending on # of available records; see Table A in S1 File), were used as background data to compare environmental attributes with presence records for our boreal species. This causes background data to display a similar selection bias as our occurrence data, allowing the model to highlight species environmental suitability within the sampled space rather than according to a sampling bias [46,47]. Given the additional sources of presence records for caribou, a target group approach would not represent the sampled space for the species, and instead we corrected for potential bias by subsampling presence records as has been previously suggested (see [48]). By correcting bias, we assume that spatial clumping of presence data for each species was not primarily based on patterns of occurrence or habitat selection for the species.

We developed MaxEnt models for all species using background records consisting of the species’ distribution and a 500km buffer, as restricted backgrounds can improve the performance of the initial model (i.e., excluding areas that have not been surveyed for the target species or that extend beyond the likely ability of the focal species to sample the environment; (see [35]). Although restricted backgrounds can increase the potential for model extrapolation into novel environments under climate change projections, this approach remains more appropriate for developing a robust initial model [35].

MaxEnt has numerous user defined settings (i.e., feature class, regularization; see [34]) that can impact model performance [49]. Species-specific tuning of the parameters is suggested to generate the most robust models [50], and this is especially important for studies that require transferring of models through space and time [51]. Here, we used the R package ENMeval, which builds a number of candidate MaxEnt models for each species with a variety of user-defined settings [51]. ENMeval further provides multiple evaluation metrics for selecting the best model. For each species, we built candidate models using every combination of feature class, while adjusting regularization values (i.e., 0.5–8). We selected the best-fit model to use in our analysis as the model that resulted in the lowest AIC. However, if there was evidence of overfitting in that model (AUC.difference >0.10; see [51]), we selected the next best fit model under that threshold. We performed a 10-fold cross-validation procedure (where 10% of presence records are set aside to test the model, and this process is repeated 10 times) to create the final MaxEnt models (see Table A in S1 File for beta-multiplier used and test AUC values for each species’ top model).

In each future projection, we included the environmental variables mentioned above, and performed a 10-fold cross-validation procedure on the best fitting model to create the MaxEnt models for each GCM for each scenario. To account for variation among GCMs, we created a final projection environmental suitability map for each species by taking the mean environmental suitability value from each separate GCM.

### b) Changes in environmental suitability

To model changes in environmental suitability, we converted continuous MaxEnt models into binary suitable/unsuitable models where values greater than the fixed cumulative value 10 (i.e., value of 10 on the cumulative output of MaxEnt) were considered suitable. This represents a ‘strict’ threshold, see [52]. For our calculations of range-wide suitability change in these species, we did not allow for northward migrations beyond the boreal treeline over the duration of our 65-year projections. Range shift models commonly assume that the northern treeline is fixed [53], given that limited colonization beyond the treeline seems to characterize most boreal tree species. For example, white spruce expansion at its northwestern range extent is limited by temperature-induced drought stress [54], whereas black spruce is challenged to become established on drought-prone tundra in the northeast [55]. Although some species like snowshoe hares recently expanded their distribution northward beyond treeline [56], such range extensions remain exceptional. Moreover, northward range expansion beyond treeline represents a small portion of the overall distribution of the species that is negligible at the scale of the boreal forest biome [56]. We calculated the change in suitable area for each species at the range-wide scale as well as within the recognized current extent of the boreal forest, as determined by Brandt [17].

Using these models, we also examined the potential level of climate-mediated niche fragmentation using the program Fragstats [57]. Here, we determined the number of climate-suitable patches, mean patch size, and mean nearest neighbour distance between patches under current, 2050 and 2080 climate conditions. Note that the metrics we used for these analyses are standard for measuring structural habitat loss and fragmentation across a range of species [13]. Although there is more recent debate surrounding the best approach for quantifying habitat fragmentation and its importance to organisms [58], here our objective was to use established methodology to quantify climate-mediated fragmentation observed in MaxEnt models generated for our 12 species of interest. This is a largely novel approach and required use of established methodology that could be applied consistently across species and at a broad spatial scale. Therefore, we consider our fragmentation analysis as an appropriate initial step in understanding broad patterns of climate-mediated range loss in the boreal forest, and below we discuss strengths and limitations of our approach. Our analyses were conducted individually on each of the 12 species of boreal-obligate organisms, and we also determined overall change in biodiversity during current and future scenarios by calculating total number of species lost or gained in each parcel.

### c) The Ontario-Québec bottleneck

We conducted a secondary analysis focused on the Ontario-Québec border, where the North American boreal forest is narrowest and climate change impacts may be especially severe. This region extends from the southern tip of James Bay, west to the shores of Lake Superior, and east by a comparable distance, into Québec. The area extends south to the northern shore of Lake Huron and is fully contained within the identified boundaries of the boreal forest. We calculated percent change in environmental suitability for all boreal species in the bottleneck region under climate change. In addition, we determined the extent that climate suitability was lost for each species in 10 randomly-selected comparably-sized regions within the boreal forest boundary (Figure D in S1 File). This effort served to develop a statistical test to determine whether the percent change in suitability within the Ontario-Québec bottleneck was more severe than on average in the boreal forest. Fragmentation statistics for the bottleneck region were computed as described above except that most species had a low number of patches, making the measurement of nearest neighbour distance less relevant.

## Results

### a) Range-wide changes in climate suitability

Our models predicted that climate change will alter niche suitability at the southern range edge for most boreal-obligate species under investigation (Table 1). For species full ranges, the area of potential occurrence will decline by a mean of –9.1 ± 8.6% (± 95% CI) (median: -13.4%) by 2050, with 9 of 12 species experiencing reduced extent of climate niche suitability (Table 1). By 2080, decline in area of potential occurrence is -19.0 ± 11.7% (median: -23.1%), with 10 species experiencing reduced extent of potential occurrence. Patterns of loss are broadly similar across taxa, with decline in niche suitability being most prominent in the southern range, especially the contiguous United States. Notably, jack pine (*Pinus banksiana*) and northern flying squirrel (*Glaucomys sabrinus*) are the only species that may experience potential improvement in climate suitability by 2080, with marked loss for flying squirrels in their southern range margins being countered by substantive gains at the northern edge (see Figure B in S1 File).

**Table 1:** Percent change in climate suitability of boreal forest species between current and future climate projections (i.e. 2050 and 2080). Changes were calculated for the full current range of each species, the current extent of the boreal forest, and the Ontario-Québec bottleneck region.

**Species**	**Full Range**		**Boreal Forest**		**Bottleneck Region**	
	2050	2080	2050	2080	2050	2080
**Trees**						
White birch (*Betula papyrifera*)	-1.1	-3.2	15.8	16.3	0.0	-0.5
White spruce (*Picea glauca*)	-16.7	-28.8	-9.2	-21.7	-5.4	-87.0
Black spruce (*Picea mariana*)	-9.6	-22.1	-2.5	-13.6	-1.6	-72.0
Jack pine (*Pinus banksiana*)	10.1	14.5	21.2	26.8	0.0	0.0
**Birds**						
Spruce grouse (*Falcipennis canadensis*)	-20.5	-32.5	-17.1	-30.1	-89.0	-100
Gray jay (*Perisoreus canadensis*)	-16.0	-24.1	-9.5	-18.6	-14.3	-83.8
Boreal chickadee (*Poecile hudsonicus*)	-21.9	-44.4	-17.9	-40.4	-61.2	-100
**Mammals**						
Moose (*Alces alces*)	-20.7	-42.6	-14.7	-36.7	-72.2	-100
Northern Flying Squirrel (*Glaucomys sabrinus*)	25.4	16.9	32.4	31.4	0.0	0.0
Snowshoe hare (*Lepus americanus*)	-10.8	-12.5	-1.0	-1.4	-0.4	-2.0
Marten (*Martes americana*)	-0.6	-9.3	8.2	-1.1	-33.7	-71.2
Caribou (*Rangifer tarandus*)	-26.5	-39.6	-38.3	-54.8	-100	-100

When the analysis was restricted to the boundaries of the boreal biome rather than species distributions, we noted a similar trend, with most boreal species losing predicted climate suitability in the southern portion of the biome (see Figure B in S1 File). Models show that by 2050, mean loss in the area of potential occurrence is –2.7 ± 11.1% (median: -5.9%; Table 1), and by 2080 this loss increases to –12.0 ± 15.4% (median: -16.1%). As with the reported changes corresponding to species distribution, gains in climate suitability within the boreal forest mainly reflect improved climate niche suitability at the northern limits (Figure B in S1 File).

### b) Climate-mediated fragmentation

**Fig 1:**
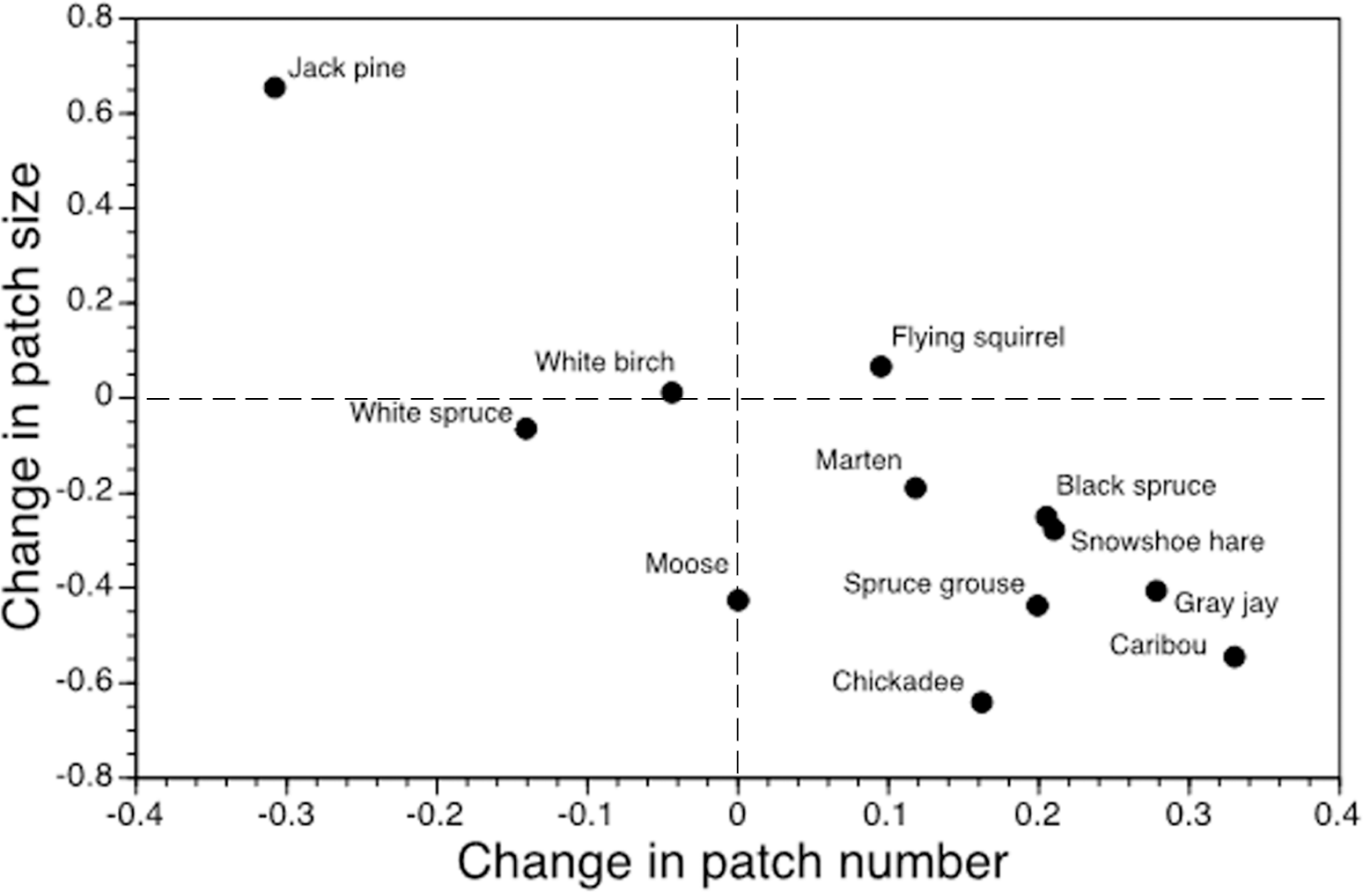
Dynamics of climate-mediated fragmentation of suitable patches for 12 boreal-obligate species. The units are proportional change in range-wide patch number relative to proportional change in patch size (2080). Zero change reflects stationary dynamics, positive values reflect increase, and negative values represent decrease.

For most species, the spatial structure of suitable climate niches will be altered substantially under climate change. When the analysis was restricted to current species distributions, number of suitable patches increased by an average of +18.8 ± 25.3% (median: +25.5%) and +26.7 ± 28.7% (median: +34.0%) by 2050 and 2080, respectively (Table 2). Jack pine, white birch (*Betula papyrifera*), and white spruce (*Picea glauca*) were the only species with fewer suitable patches by 2080, and in the case of jack pine and white birch, the lower number of patches reflects improved climate suitability between existing suitable patches and consolidation of currently-disjunct patches; for white spruce, reduced number of suitable patches is due to marked loss of climate suitability in the southern range (Table 1). On average, mean patch size could decline by -13.3 ± 18.2% (median: -22.0%) and -22.6 ± 19.7% (median: -31.5%) by 2050 and 2080, respectively (Table 2). Overall, there is a strong negative relationship between number and size of climate-suitable patches, with only flying squirrel experiencing an increase in both number and size of climate-suitable patches (Fig 1). Isolation between suitable patches also will increase under climate change, with a mean increase in inter-patch distance averaging +2.4 ± 3.3% and +5.8 ± 4.2% by 2050 and 2080, respectively (see Table 3).

**Table 2:** Number of suitable patches and mean patch size for boreal forest species under current and future climate projections (2050, 2080), within the full current range of each species.

**Species**	**Number of patches**			**Mean patch size (km**^**2**^**)**		
	Current	2050	2080	Current	2050	2080
**Trees**						
White birch (*Betula papyrifera*)	252	274	241	35923.8	32686.5	36344.8
White spruce (*Picea glauca*)	192	171	165	42447.4	39715.2	35170.3
Black spruce (*Picea mariana*)	156	188	188	51849.4	38888.8	33517.0
Jack pine (*Pinus banksiana*)	253	180	175	26141.9	40436.1	43269.7
**Birds**						
Spruce grouse (*Falcipennis canadensis*)	267	294	320	30338.6	21893.5	17087.2
Gray jay (*Perisoreus canadensis*)	259	290	331	35207.7	26430.0	20907.3
Boreal chickadee (*Poecile hudsonicus*)	265	324	308	34962.6	16780.6	12564.0
**Mammals**						
Moose (*Alces alces*)	146	230	146	51052.0	25685.2	29312.3
Northern Flying Squirrel (*Glaucomys sabrinus*)	316	289	346	24474.37	33549.5	26119.4
Snowshoe hare (*Lepus americanus*)	238	262	288	42126.89	34117.9	30472.6
Marten (*Martes americana*)	304	298	340	24604.3	24941.3	19948.5
Caribou* (*Rangifer tarandus*)	364	437	484	24005.0	14688.8	10911.6

* Includes subspecies beyond the northern treeline

**Table 3:** Percent change in nearest neighbor distance for boreal forest species between current and future climate projections (2050, 2080), within the full range of each species and the boreal forest.

**Species**	**Full range**			**Boreal Forest**		
		2050	2080		2050	2080	
**Trees**							
White birch (*Betula papyrifera*)		-1.11	11.3		7.9	-8.5	
White spruce (*Picea glauca*)		5.6	0.9		2.9	2.1	
Black spruce (*Picea mariana*)		11.5	5.6		-2.6	7.0	
Jack pine (*Pinus banksiana*)		4.2	21.7		-3.2	3.1	
**Birds**							
Spruce grouse (*Falcipennis canadensis*)		-0.9	-3.4		16.1	5.3	
Gray jay (*Perisoreus canadensis*)		1.9	2.0		9.0	10.7	
Boreal chickadee (*Poecile hudsonicus*)		0.6	1.3		-5.4	-1.6	
**Mammals**							
Moose (*Alces alces*)		-11.9	0.3		9.9	15.4	
Northern Flying Squirrel (*Glaucomys sabrinus*)		8.9	15.1		2.2	-0.3	
Snowshoe hare (*Lepus americanus*)		1.4	2.9		-3.1	-4.4	
Marten (*Martes americana*)		5.9	10.4		-4.8	-2.8	
Caribou (*Rangifer tarandus*)		3.3	1.0		9.5	9.2	

When the analysis was restricted to the boreal forest region [17], we noted marked changes in level of fragmentation among climate-suitable patches. On average, number of patches increased by +13.4 ± 29.0% (median: +7.5%) and +22.3 ± 38.3% (median: +27.0%) for 2050 and 2080, respectively, with 8 species experiencing net increase patch number by 2080 (Table B in S1 File). By 2050, mean patch size increased by +0.9 ± 32.8% (median: -16.0%) but only five species experienced patch size increase (Table B in S1 File), reflecting northward range shift for species currently having more restricted northern range extent. By 2080, a smaller increase (+0.4 ± 48.0% (median: -28.7%)) in mean patch size was observed. Notably, jack pine could experience a nearly 200% increase in mean patch size by 2080, whereas mean patch size could decline for 8 species (Table B in S1 File). Nevertheless, our projections support that climate-mediated fragmentation will become increasingly important in the boreal forest: By 2080, 8 species should experience increased number of suitable patches combined with reduced mean patch size. Furthermore, most species will experience increased distance between climate-suitable patches, with mean distance increase averaging +3.2 ± 4.0% and +2.9 ± 3.9% by 2050 and 2080, respectively (Table 3).

### c) Changes in the Ontario-Québec bottleneck

Our models predict a substantive loss of climate-suitable niche space in the Ontario-Québec bottleneck region. Suitable space could decrease by an average of -31.5 ± 21.8% (median: -9.8%) and -59.7 ± 25.3% (median: -77.9%) by 2050 and 2080, respectively (Table 1). By 2050, only white birch, jack pine and northern flying squirrel could fully retain complete suitability in the bottleneck region; this pattern is reduced to only jack pine and flying squirrel by 2080.

Caribou likely will experience the most pronounced decrease in climate suitability in the bottleneck region, with 100% loss of suitable space predicted by 2050. However, by 2080 boreal chickadee (*Poecile hudsonicus*), moose (*Alces alces*) and spruce grouse (*Falcipennis canadensis*) also could be extirpated from the region (Table 1). Black spruce (*Picea mariana*), gray jay (*Perisoreus canadensis*), marten (*Martes americana*) and white spruce each could experience >70% decline in climate suitability by 2080.

For all 12 species, the bottleneck region will host greater loss in climate suitability compared to changes observed among random sites across the boreal forest. Reductions in area of potential occurrence averaged -28.7 ± 27.7% (median: -19.3%) and -47.1 ± 29.3% (median: -54.1%) less than the random sites by 2050 and 2080, respectively (Table 4). For both 2050 and 2080, all species will experience a decrease in climate suitability in the bottleneck region, compared to random sites (Table 4).

**Table 4:** Change in climate suitability (%) for boreal forest species in randomly-selected regions of the boreal forest and the Ontario-Québec bottleneck region.

**Species**	**2050**		**2080**	
	Random	Bottleneck	Random	Bottleneck
**Trees**				
White birch (*Betula papyrifera*)	7.6 ± 10.8	0.0	8.4 ± 12.2	-0.5
White spruce (*Picea glauca*)	-0.63 ± 1.0	-5.4	-7.5 ± 5.0	-87.0
Black spruce (*Picea mariana*)	0.2 ± 0.6	-1.6	-3.4 ± 2.9	-72.0
Jack pine (*Pinus banksiana*)	8.5 ± 9.1	0.0	10.7 ± 12.1	0.0
**Birds**				
Spruce grouse (*Falcipennis canadensis*)	-11.0 ± 8.4	-89.0	-31.3 ± 15.0	-100
Gray jay (*Perisoreus canadensis*)	-0.6 ± 0.6	-14.3	-7.3 ± 7.6	-83.8
Boreal chickadee (*Poecile hudsonicus*)	-5.2 ± 7.9	-61.2	-34.8 ± 17.0	-100
**Mammals**				
Moose (*Alces alces*)	-4.9 ± 4.0	-72.2	-29.1 ± 14.9	-100
Northern Flying Squirrel (*Glaucomys sabrinus*)	17.4 ± 16.8	0.0	17.7 ± 17.1	0.0
Snowshoe hare (*Lepus americanus*)	0.2 ± 0.5	-0.4	0.2 ± 0.5	-2.1
Marten (*Martes americana*)	10.0 ± 10.8	-33.7	6.3 ± 13.3	-71.2
Caribou (*Rangifer tarandus*)	-43.8 ± 15.1	-100	-68.7 ± 16.5	-100

Our models predict that under climate change, the bottleneck region will become increasingly fragmented for most boreal species. Eight species may experience either complete loss of suitability or increased number of habitat patches by 2050, while 9 species could experience either complete loss of suitability or reduction in mean suitable patch size by 2080 (Table 1, Table C in S1 File).

### d) Changes in species richness

**Fig 2:**
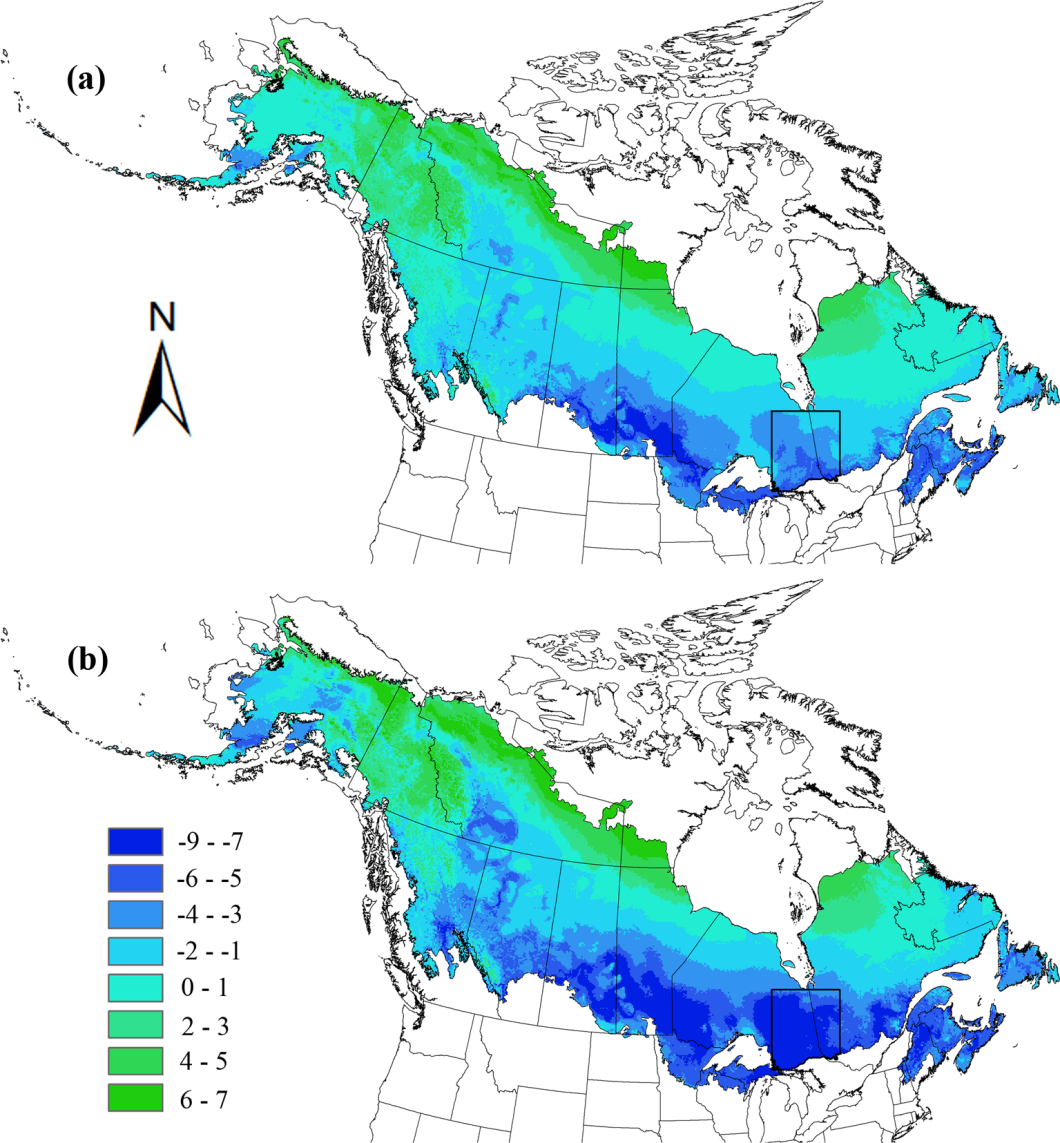
Predicted change in diversity of 12 boreal-obligate species in 2050 (a) and 2080 (b). Dark blue represents areas where species diversity decreased under climate change whereas light blue indicates zero change. Green color indicates a positive change in diversity of the 12 species.

By 2080, richness of boreal-obligate species will decline, with such decline being most dramatic in the bottleneck region (Fig 2). On average, predicted change in species across the entire boreal region is -1.35 of 12 species, compared to more than half (i.e., -6.93) of 12 species when the analysis is restricted to the bottleneck region. In contrast, the predicted decline in 10 randomly-located bottleneck areas is -1.49 ± 1.14 of 12 species. Results are less severe, but similar in pattern, when analyzing projected species richness in 2050. Predicted change across the boreal is -0.34 of 12 species, compared to -3.54 in the bottleneck (species declines in 10 randomly located bottleneck areas is -0.33 ± 0.29). In addition to loss of boreal-obligate species richness in the Ontario-Québec bottleneck region, major species declines are predicted across the southern boreal and mixed forest region, including in Maine, Nova Scotia, New Brunswick, southeastern Québec, southwestern Ontario, northern Michigan, northern Minnesota, southern Manitoba, southern Saskatchewan, and south-central Alberta (Fig 2).

## Discussion

We report that, for a variety of species in the boreal forest, loss of climate suitability will cause a striking pattern of predicted range size reduction and shift. For many species, the next 65 years may herald substantive southern range recession that is only partially offset by increased suitability and potential expansion in the northern range. Our models also predict marked fragmentation of many species ranges, with increased number, smaller size, and greater isolation of suitable patches, leading to overall lower quality of remaining patches. This phenomenon will be most severe in the core boreal region near the Ontario-Québec border, where several species likely will lose all climate suitability, resulting in disjunct east-west population segments. Although the prediction that species will experience substantive climate-mediated range loss and range shift is not new, including for boreal species [59], we provide novel forecasts concerning the extent of large-scale discontinuity in species distributions that will lead to fundamental change in meta-population dynamics, gene flow, and species richness (see [60]). To date, insight into large-scale climate-mediated range fragmentation across a variety of taxa has not received concerted attention, although recent studies have attempted to identify such outcomes in birds [61,62]. Accordingly, our findings provide a basis for future work assessing changes in ecosystem structure and composition across the boreal biome in North America.

### a) Climate-mediated fragmentation

It is widely recognized that the boreal forest will be strongly influenced by the effects of climate change, as noted previously by studies reporting reduced species distribution and abundance [63], northward (or elevational) shift in occurrence [59], phenological mismatch [64], and altered ecosystem structure and function [20]. Here, we provide evidence that climate change may impose additional costs via fragmentation of species’ ranges that will take place at a large spatial scale and involve a variety of species, such that the integrity of the boreal biome itself could be dramatically compromised in these regions. To our knowledge, ours is the first study to explicitly differentiate between climate-mediated species range loss versus climate-mediated range fragmentation, and given this novelty and the potential implications of our findings it is important to evaluate how climate may fragment species distributions and whether these predictions are supported by known species relationships with their environment.

First, we invoke the process of habitat fragmentation, traditionally defined as the breaking apart of continuous patches of suitable habitat into smaller patches [13], to help infer how climate change may fragment otherwise suitable environments. Habitat fragmentation is a landscape-level process that results in: 1) increased number of suitable habitat patches, 2) reduced size of suitable patches; 3) increased distance between suitable patches, and; 4) increased edge-to-area ratio of suitable patches [13]. There is an extensive body of both theoretical and empirical literature showing that habitat fragmentation is a common outcome of landscape deterioration, usually driven by anthropogenic activities [16,65]. While both theoretical and empirical literature suggests that the direct effects of habitat loss normally outweigh those of habitat fragmentation [66,67], this is not always the case [65,68,69], and the effects of habitat fragmentation may be most severe when amount of suitable habitat is limited [70,71]. Fragmentation often exerts strong independent effects on population connectivity [72,73], although precisely quantifying the effects of fragmentation is challenging because habitat loss and fragmentation are often highly correlated [74,75], and there is an incomplete state of knowledge regarding the influence of habitat fragmentation alone.

We contend that climate change should elicit patterns of landscape fragmentation and attendant changes in species ranges that reasonably comply with those elicited by anthropogenic habitat fragmentation. This is supported by the larger number of environmentally-suitable patches, smaller size of patches, and greater spatial isolation of patches, predicted by our analysis. Although these changes are driven in part by correlations with loss of area of suitable climate, species ranges are also independently experiencing fragmentation of climate niches to varying degrees at multiple scales, which highlights the necessity of considering both processes when examining impacts of future climate change on species persistence. For example, our models demonstrated that the boreal forest range of boreal chickadee and moose were reduced in area by a similar amount by 2080, but boreal chickadees experienced higher levels of fragmentation, with almost double the increase in the number of patches. At a large spatial scale, we forecast that fragmentation in the Ontario-Québec bottleneck in particular will lead to range discontinuity and regional extirpation or marked decline in distribution and abundance of many boreal species. Such effects will have profound implications for conservation of the structure and integrity of the boreal forest of North America.

However, we recognize that there are important differences between traditional habitat fragmentation versus climate-mediated fragmentation that could influence the relevance and interpretation of fragmentation analyses such as ours. In particular, climate-mediated fragmentation will mainly occur at a regional or continental scale, and therefore will correspond to changes at the extent of broad climate zones rather than at the scale of land parcels or administrative jurisdictions, which is typical of traditional habitat fragmentation. This means that boundaries associated with climate-mediated fragmentation will reflect gradual spatial (or temporal) environmental transition and therefore appear as being more diffuse on the landscape compared to the sharp edges normally caused by anthropogenic habitat fragmentation. Accordingly, the increasing amount of edge in fragmented landscapes, which plays an important role in altering biodiversity in traditional fragmentation analyses [65], may be less relevant when quantifying climate-mediated fragmentation. Likewise, landscape variegation occurring at a small scale and resulting in a fine-resolution checkerboard of habitat suitability also may be less important. Regardless, we foresee an overall strong similarity in the processes and outcomes of climate-mediated fragmentation versus those from traditional habitat fragmentation, meaning that there should be a common foundation for understanding the extent and magnitude of fragmentation-type responses in changing environments. For example, limited evidence suggests that fragmentation of suitable climate may have similar effects on population connectivity as would be expected from traditional habitat fragmentation, limiting dispersal and increasing genetic differentiation [15,76–78]. Importantly, the effects of climate-mediated fragmentation on connectivity may be much more difficult to mitigate than effects of habitat fragmentation. Indeed, although habitat corridors are often proposed to reduce negative effects of habitat-mediated fragmentation [75], comparable corridors having suitable climate space may not exist and cannot be created [78].

### b) Climate change and species responses

Our study involved 12 boreal species, spanning diverse taxa and with a range of interactions with climate and the environment. Thus, predicted responses to climate change should be generalizable to a variety of species that rely on the boreal forest (see [29]). Interestingly, the high repeatability of predicted range variation across species illustrates that despite high taxonomic diversity, common factors ultimately will drive species responses to climate change. Our predictive models highlight both mean temperature, and maximum temperature of the warmest month, as being especially important drivers across many species (Table D in S1 File), and these correlated variables are general indicators of climate change trends that can underlie both direct and indirect effects of climate variability on species occurrence. For instance, white spruce face temperature-induced drought [54] whereas moose may experience heat conditions that exceed thermoregulatory thresholds [79]; both phenomena reflect direct effects of climate change that can lead to variation in the distribution and abundance of climate-sensitive species. In contrast, gray jays require cold temperatures to maintain winter food caches [80] whereas snowshoe hares experience higher predation risk with receding snow cover [64]. In both cases, climate-related effects are indirect through correlated biotic processes (i.e., food availability, predation) that are themselves associated with climate. Yet, there is debate regarding the importance of biotic interactions at large spatial scales [81,82], as well as specifically in the context of species distribution models [83], although we note that recent work (e.g., [38,39]) highlights that the MaxEnt approach yields models that closely match observed patterns of species occupancy and population growth. Therefore, we suggest that our models represent a reasonable first step in understanding how species occurrence may vary through space and time under climate change, despite that they exclude fundamental biotic processes [82]. That our predicted responses are remarkably consistent across many boreal species reinforces this contention while also implying that singular climate phenomena should transcend taxonomic groups and life histories to affect distribution and abundance for an array of species.

### c) Future research directions

Climate change can impose strong directional selection, leading to changes in gene frequencies and allowing for local adaptation (e.g., [84]). Further, phenotypic plasticity can track environmental variability under rapid climate change (e.g., [85]). Yet, while genetic shifts or plasticity may modulate local effects of climate change [86], all environmental niche models assume that the climate-species relationship is static and that future species distributions reflect current species-environment relationships. In addition, genetic or phenotypic responses to climate change are unlikely to be sufficient to mitigate negative responses at the species level ([87],but see [88]), making it unrealistic that all species will have comparable ability to adapt to rapidly changing environments. However, uncertainty in the magnitude, timing, and spatial extent of climate adaptation can serve to develop explicit predictions that can be tested empirically. For example, we predict that the Ontario-Québec bottleneck region will become key for climate-driven phenotypic plasticity and selection. In fact, there is already evidence of climate-mediated genetic separation in this area [14,15], providing indirect support that distinct east-west population segments will arise for some species. As a logical outcome of our models, we predict greater expression of phenotypic plasticity and disparity in climate-related gene frequencies (e.g., resistance to cold or drought) in areas with rapid and substantive changes in climate suitability, although the extent to which directional selection will prevail over local extinction requires much more analysis. Regardless, an improved understanding of such responses from field observations, guided by our spatially-explicit model predictions, will allow researchers to determine the extent that local adaptation may be driven by climate change.

Our results also highlight hotspots for extensive species loss and ultimately, major shifts in boreal forest ecosystems. It follows that these areas also will be especially vulnerable to species invasions and cascading effects of such invasions through widespread changes in ecosystem structure and function. For example, increasing winter temperatures and attendant changes in freeze-thaw dynamics in the Ontario-Québec bottleneck should promote recession of spruce in the region and facilitate expansion of more drought-tolerant tree species [89]. Likewise, decline in moose and caribou in the region could elicit commensurate decline in their native predators like wolves (*Canis lupus*) and facilitate colonization of vacant niches by invading coyotes (*Canis latrans*) or wolf-coyote hybrids [90,91]. Yet, such changes are especially difficult to forecast in complex environments, but they do provide an important opportunity to develop a research program tailored to better understand ecosystem-level responses in climate change hotspots. To date, similar work has barely been initiated but will prove crucial for improving our ability to functionally explain, forecast, and perhaps mitigate, large-scale climate change effects in the boreal and other biomes.

## Supporting information

S1 File Table A presents the number of presence records, target group, beta-multiplier, and feature types used for the top MaxEnt models developed for each species. Table B describes the number of suitable patches and mean patch size for boreal forest species under current and future climate projections (2050, 2080), within the current distribution of the boreal forest. Table C includes the number of suitable patches and mean patch size for boreal forest species under current and future climate projections (2050, 2080), within the Ontario-Québec bottleneck region. Table D provides contributions of environmental variables to climate niche suitability of boreal forest species. Note that the listed variables represent those contributing most to MAXENT models but they may not drive actual niche suitability and therefore caution is warranted in interpretation. Figure A provides distribution of presence records for 12 boreal-obligate trees, birds and mammals. Figure B describes change in environmental suitability for 12 boreal-obligate species within the boreal biome from current to 2080. Red represents lost suitability, blue represents suitability gain, and green indicates currently suitable cells that remain suitable. Solid line represents the Ontario-Québec bottleneck region. Figure C includes change in climate between current and 2080 for environmental variables used to generate climate suitability models. Future climate data used in the calculation for these figures were created by averaging values between the two projection sources (CGCM3, CSIRO mk3.5). Figure D describes replicates of the Ontario-Québec bottleneck region. The solid line represents the actual bottleneck and the extent of the boreal forest, and dotted lines represent randomly-selected replicates.
(DOCX)
