# Supplementary material for: Continental divide: Predicting climate-mediated fragmentation and biodiversity loss in the boreal forest
Source: PLoS One. 2017 May 15;12(5):e0176706. doi: 10.1371/journal.pone.0176706 (PMC5432165; doi:10.1371/journal.pone.0176706)
Supplement: S1 File — Table A presents the number of presence records, target group, beta-multiplier, and feature types used for the top MaxEnt models developed for each species. Table B describes the number of suitable patches and mean patch size for boreal forest species under current and future climate projections (2050, 2080), within the current distribution of the boreal forest. Table C includes the number of suitable patches and mean patch size for boreal forest species under current and future climate projections (2050, 2080), within the Ontario-Québec bottleneck region. Table D provides contributions of environmental variables to climate niche suitability of boreal forest species. Note that the listed variables represent those contributing most to MAXENT models but they may not drive actual niche suitability and therefore caution is warranted in interpretation. Figure A provides distribution of presence records for 12 boreal-obligate trees, birds and mammals. Figure B describes change in environmental suitability for 12 boreal-obligate species within the boreal biome from current to 2080. Red represents lost suitability, blue represents suitability gain, and green indicates currently suitable cells that remain suitable. Solid line represents the Ontario-Québec bottleneck region. Figure C includes change in climate between current and 2080 for environmental variables used to generate climate suitability models. Future climate data used in the calculation for these figures were created by averaging values between the two projection sources (CGCM3, CSIRO mk3.5). Figure D describes replicates of the Ontario-Québec bottleneck region. The solid line represents the actual bottleneck and the extent of the boreal forest, and dotted lines represent randomly-selected replicates. (DOCX) [file pone.0176706.s001.docx]

**S1 File**

**Table A:** Number of presence records, target group, beta-multiplier, and feature types used for the top MaxEnt models developed for each species.

| Species | # of Records | Target group | Beta-multiplier | Feature Type^a^ |
| --- | --- | --- | --- | --- |
| Trees |  |  |  |  |
| White birch  (*Betula papyrifera*) | 621 | Fagales | 1 | LQP |
| White spruce  (*Picea glauca*) | 478 | Pinales | 1 | H |
| Black spruce  (*Picea mariana*) | 404 | Pinales | 1.5 | H |
| Jack pine  (*Pinus banksiana*) | 245 | Pinales | 3 | LQP |
| Birds |  |  |  |  |
| Spruce grouse  (*Falcipennis canadensis*) | 636 | Phasianidae | 1.5 | H |
| Gray jay  (*Perisoreus canadensis*) | 2238 | Corvidae | 2.5 | H |
| Boreal chickadee  (*Poecile hudsonicus*) | 497 | Paridae | 1 | LQP |
| Mammals |  |  |  |  |
| Moose  (*Alces alces*) | 1511 | Artiodactyla | 0.5 | H |
| Northern Flying Squirrel  (*Glaucomys sabrinus*) | 458 | Sciuridae | 1 | LQ |
| Snowshoe hare  (*Lepus americanus*) | 320 | Lagomorpha | 1 | LQP |
| Marten  (*Martes americana*) | 554 | Carnivora | 0.5 | H |
| Caribou  (*Rangifer tarandus*) | 1629 | Subsample | 1.5 | H |

^a^Feature types used in the model: H = Hinge, LQ = Linear and Quadratic, LQP = Linear, quadratic, and product

**Table B**: Number of suitable patches and mean patch size for boreal forest species under current and future climate projections (2050, 2080), within the current distribution of the boreal forest.

| Species | Number of patches | | | Mean patch size (km^2^) | | |
| --- | --- | --- | --- | --- | --- | --- |
|  | **Current** | **2050** | **2080** | **Current** | **2050** | **2080** |
| Trees |  |  |  |  |  |  |
| White birch  (*Betula papyrifera*) | 103 | 80 | 71 | 60486.41 | 90142.50 | 102054.93 |
| White spruce  (*Picea glauca*) | 76 | 107 | 121 | 92539.47 | 59675.70 | 45525.62 |
| Black spruce  (*Picea mariana*) | 83 | 68 | 65 | 82437.35 | 98086.76 | 90984.62 |
| Jack pine  (*Pinus banksiana*) | 180 | 113 | 77 | 28737.22 | 55484.07 | 85170.13 |
| Birds |  |  |  |  |  |  |
| Spruce grouse  (*Falcipennis canadensis*) | 93 | 103 | 137 | 73215.05 | 54776.70 | 34755.47 |
| Gray jay  (*Perisoreus canadensis*) | 82 | 106 | 118 | 88735.37 | 62125.47 | 50209.32 |
| Boreal chickadee  (*Poecile hudsonicus*) | 127 | 240 | 258 | 48659.06 | 21140.83 | 14282.95 |
| Mammals |  |  |  |  |  |  |
| Moose  (*Alces alces*) | 61 | 142 | 135 | 107365.57 | 39328.17 | 30697.78 |
| Northern Flying Squirrel  (*Glaucomys sabrinus*) | 128 | 80 | 79 | 42771.88 | 90621.25 | 91068.35 |
| Snowshoe hare  (*Lepus americanus*) | 80 | 85 | 84 | 91151.25 | 84914.12 | 85635.71 |
| Marten  (*Martes americana*) | 121 | 120 | 139 | 48996.69 | 53451.67 | 42164.75 |
| Caribou  (*Rangifer tarandus*) | 148 | 199 | 265 | 39214.86 | 17989.95 | 9906.04 |

**Table C**: Number of suitable patches and mean patch size for boreal forest species under current and future climate projections (2050, 2080), within the Ontario-Québec bottleneck region.

| Species | Number of patches | | | Mean patch size (km^2^) | | |
| --- | --- | --- | --- | --- | --- | --- |
|  | **Current** | **2050** | **2080** | **Current** | **2050** | **2080** |
| Trees |  |  |  |  |  |  |
| White birch  (*Betula papyrifera*) | 1 | 1 | 1 | 289000.00 | 289000.00 | 287600.00 |
| White spruce  (*Picea glauca*) | 1 | 3 | 7 | 289000.00 | 91166.67 | 5371.43 |
| Black spruce  (*Picea mariana*) | 1 | 2 | 8 | 289000.00 | 142150.00 | 10125.00 |
| Jack pine  (*Pinus banksiana*) | 1 | 1 | 1 | 289000.00 | 289000.00 | 289000.00 |
| Birds |  |  |  |  |  |  |
| Spruce grouse  (*Falcipennis canadensis*) | 1 | 3 | 0 | 289000.00 | 10600.00 | 0 |
| Gray jay  (*Perisoreus canadensis*) | 1 | 2 | 5 | 289000.00 | 123900.00 | 9380.00 |
| Boreal chickadee  (*Poecile hudsonicus*) | 1 | 9 | 0 | 285900.00 | 12333.33 | 0 |
| Mammals |  |  |  |  |  |  |
| Moose  (*Alces alces*) | 1 | 13 | 0 | 289000.00 | 6176.92 | 0 |
| Northern Flying Squirrel  (*Glaucomys sabrinus*) | 1 | 1 | 1 | 289000.00 | 289000.00 | 289000.00 |
| Snowshoe hare  (*Lepus americanus*) | 1 | 1 | 1 | 289000.00 | 288000.00 | 283200.00 |
| Marten  (*Martes americana*) | 1 | 8 | 6 | 289000.00 | 23950.00 | 13866.67 |
| Caribou  (*Rangifer tarandus*) | 3 | 0 | 0 | 74966.67 | 0 | 0 |

**Table D.** Contributions of environmental variables to climate niche suitability of boreal forest species. Note that the listed variables represent those contributing most to MAXENT models but they may not drive actual niche suitability and therefore caution is warranted in interpretation.

| Species | Highest contributing variable  (% contribution) | | Second highest contributing variable (% contribution) | |
| --- | --- | --- | --- | --- |
| Trees | |  | |  |
| White birch  *(Betula papyrifera)* | | Mean Temp (77.5) | | Temp Seasonality (8.8) |
| White spruce  *(Picea glauca)* | | Temp Seasonality (27.5) | | Mean Temp (26.2) Mean Temp (26.2) |
| Black spruce  *(Picea mariana)* | | Max T Warmest Month (41.5) | | Min T Coldest Month (29.8) Min T Coldest Month (29.8) |
| Jack pine  *(Pinus banksiana)* | | Mean Temp (36.2) | | Temp Seasonality (20.9) Temp Seasonality (20.9) |
| Birds | |  | |  |
| Spruce grouse  *(Falcipennis canadensis)* | | Mean Temp (52) | | Max T Warmest Month (38.2) |
| Gray jay  *(Perisorius canadensis)* | | Max T Warmest Month (56.7) | | Min T Coldest Month (31.5) Min T Coldest Month (31.5) |
| Boreal chickadee  *(Poecile hudsonicus)* | | Mean Temp (45.7) | | Min T Coldest Month (43) Min T Coldest Month (43) |
| Mammals | |  | |  |
| Moose  *(Alces alces)* | | Min T Coldest Month (41.1) | | Max T Warmest Month (32.9) |
| Northern flying squirrel  *(Glaucomys sabrinus)* | | Precip Wettest Quarter (42.6) | | Mean Temp (21.8) Mean Temp (21.8) |
| Snowshoe hare  *(Lepus americanus)* | | Mean Temp (34.2) | | Min T Coldest Month (26.6) Min T Coldest Month (26.6) |
| Marten  *(Martes americanus)* | | Max T Warmest Month (57.4) | | Mean Temp (20.4) Mean Temp (20.4) |
| Caribou  *(Rangifer tarandus)* | | Mean Temp (62.8) | | Precip Wettest Quarter (13.3).3) |

**
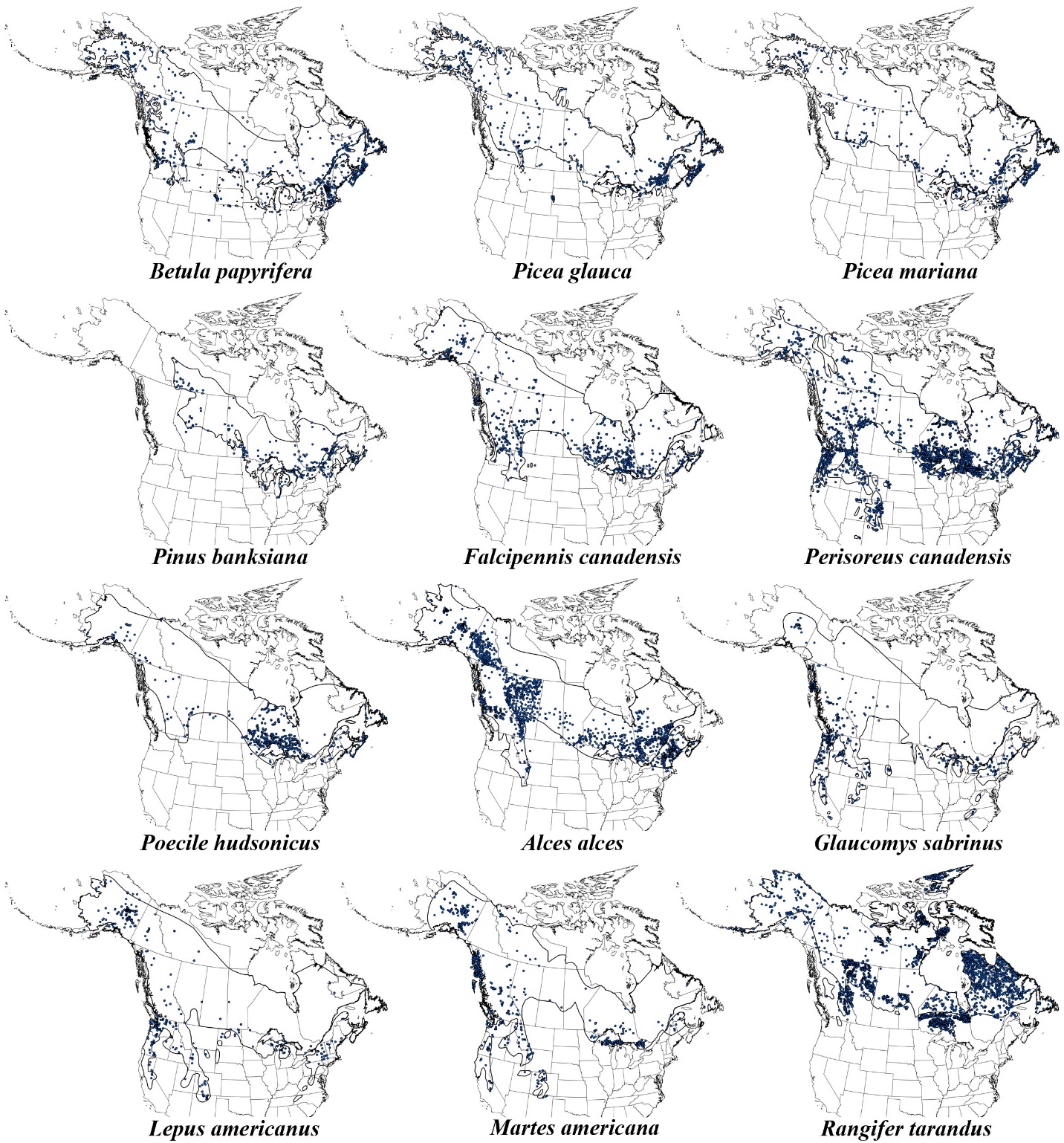
**

**Figure A:** Distribution of presence records for 12 boreal-obligate trees, birds and mammals.

**
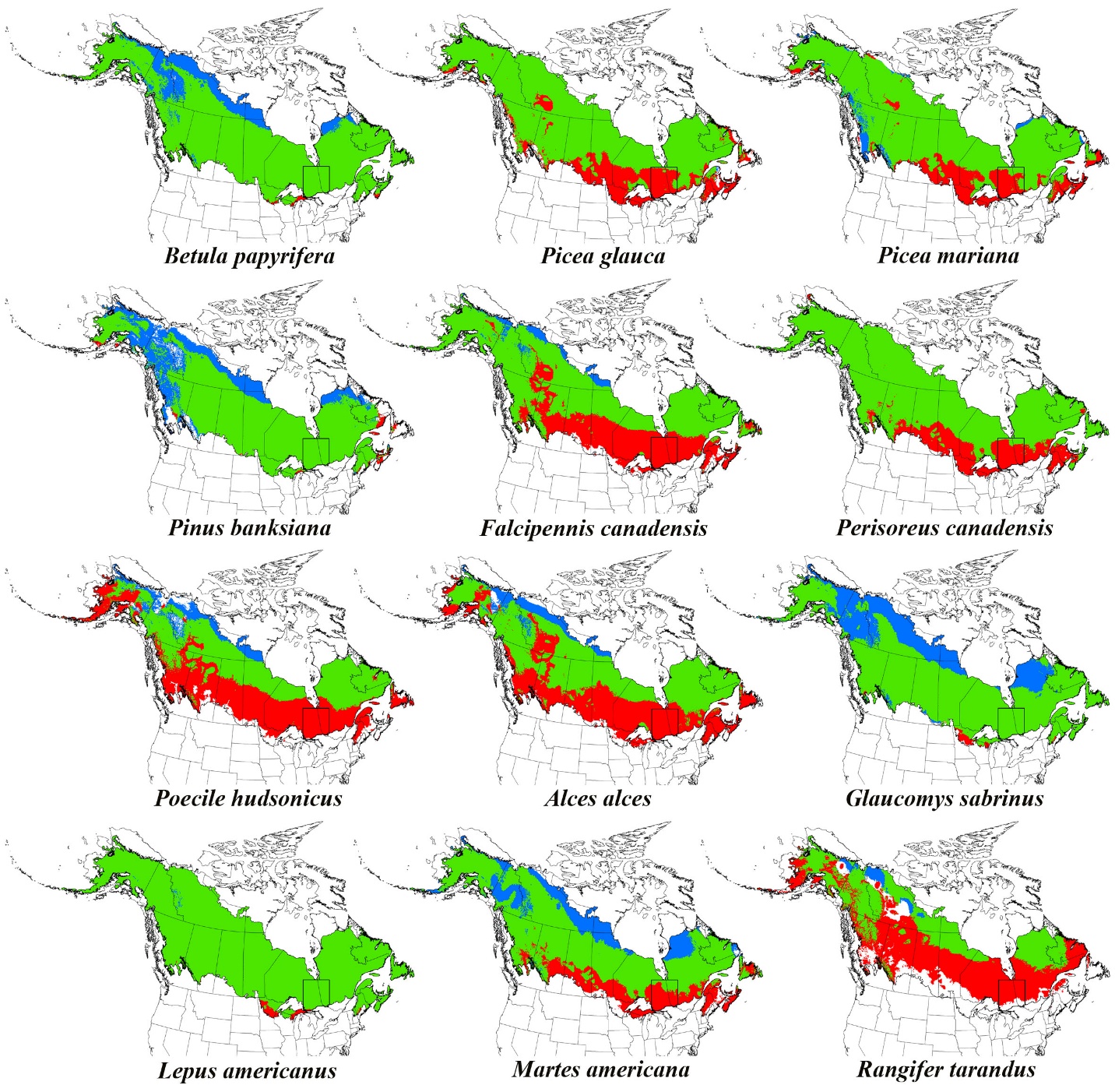
**

**Figure B:** Change in environmental suitability for 12 boreal-obligate species within the boreal biome from current to 2080. Red represents lost suitability, blue represents suitability gain, and green indicates currently suitable cells that remain suitable. Solid line represents the Ontario-Québec bottleneck region.

**
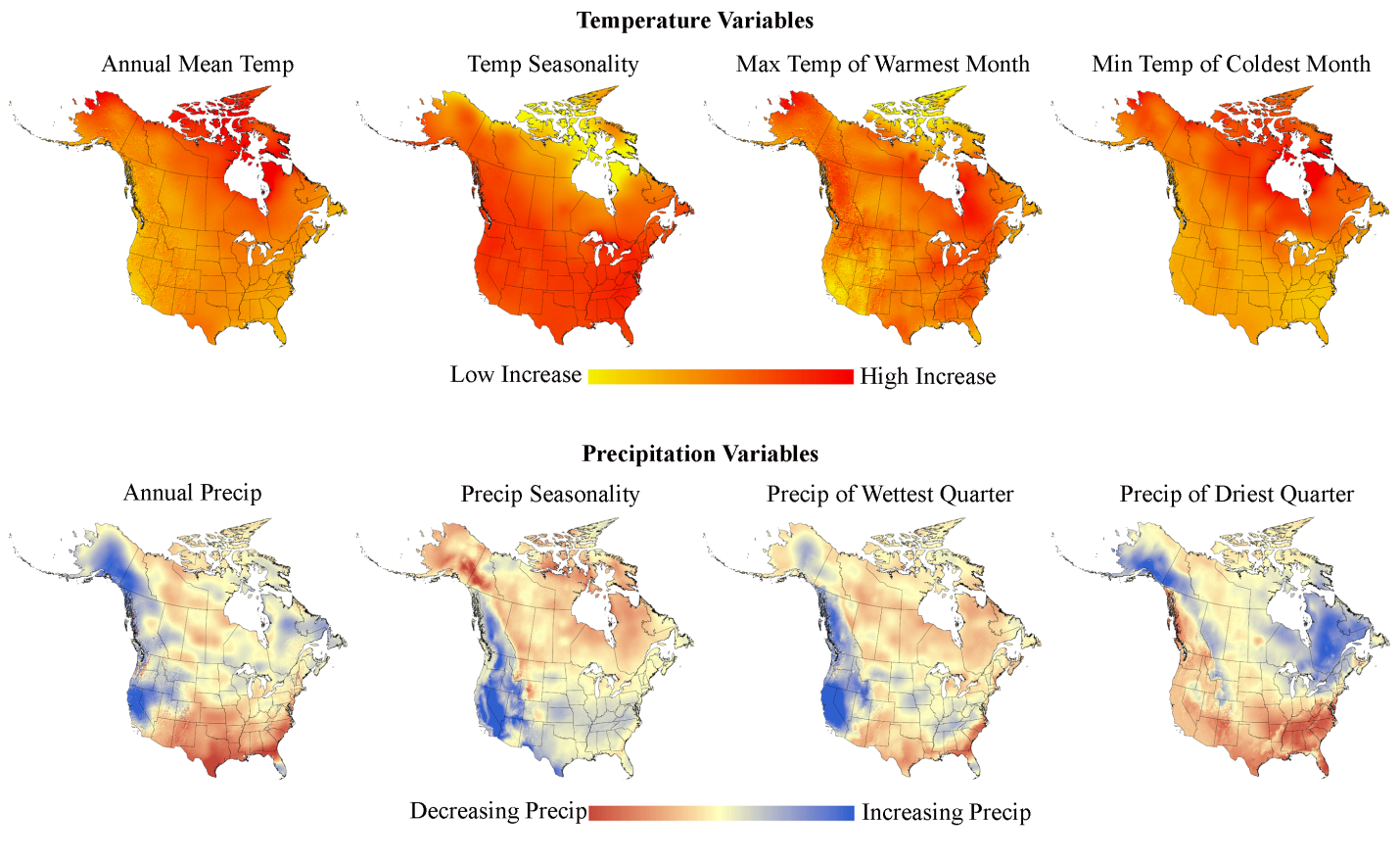
**

**Figure C:** Change in climate between current and 2080 for environmental variables used to generate climate suitability models. Future climate data used in the calculation for these figures were created by averaging values between the two projection sources (CGCM3, CSIRO mk3.5).

**
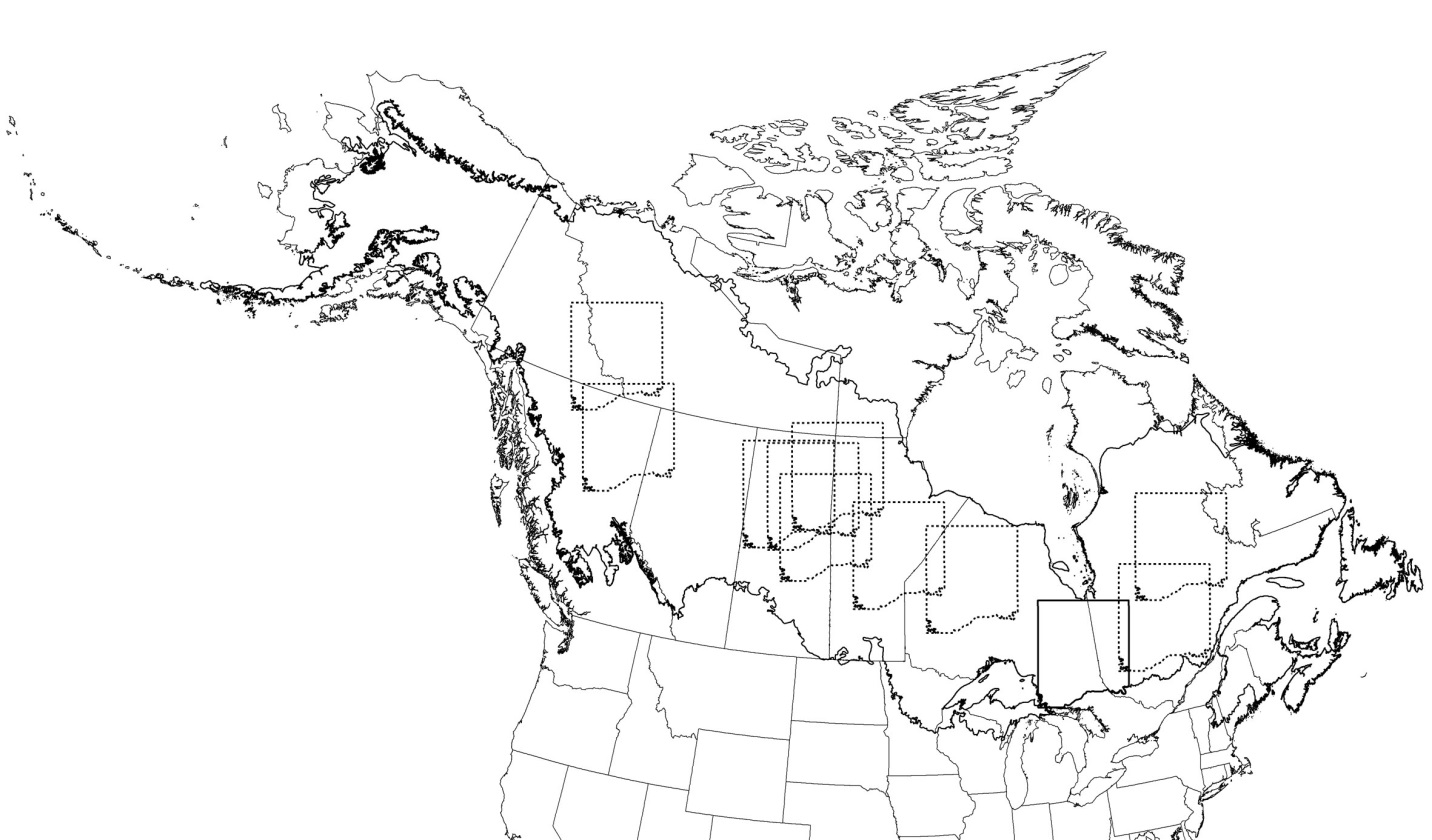
**

**Figure D**: Replicates of the Ontario-Québec bottleneck region. The solid line represents the actual bottleneck and the extent of the boreal forest, and dotted lines represent randomly-selected replicates.
